# Moment of Bentonite Addition, Co-Addition of Tannins, and Bentonite Type Affect the Differential Affinity of Pathogenesis-Related Grape Proteins towards Bentonite during Fermentation

**DOI:** 10.3390/foods9111534

**Published:** 2020-10-25

**Authors:** Igor Lukić, Ivana Horvat

**Affiliations:** 1 Institute of Agriculture and Tourism, Karla Huguesa 8, HR-52440 Poreč, Croatia; ihorvat@iptpo.hr; 2 Centre of Excellence for Biodiversity and Molecular Plant Breeding, Svetošimunska 25, HR-10000 Zagreb, Croatia

**Keywords:** wine, pathogenesis-related proteins, bentonite, fining, tannins

## Abstract

To test the effect of the moment of bentonite addition, co-addition of tannins, and bentonite type on the differential affinity of pathogenesis-related (PR) proteins towards bentonite during grape must fermentation, three separate experiments were set up. PR proteins in the obtained wines were analyzed by reverse phase and size exclusion high-performance liquid chromatography (HPLC). The most significant reduction of bentonite dose and PR protein concentration was achieved by applying bentonite in the last third of fermentation. Particular thaumatin-like proteins (TLP) and proteins with lower molecular mass in general were more affected than others, while TLPs were more affected than chitinases. Exogenous enological tannins interacted with particular PR proteins, mostly TLPs, and lowered the total bentonite dose required. The combined application of tannins and bentonite in fermentation removed more PR proteins than bentonite alone, but did not achieve a synergistic effect in reducing the bentonite dose. Various bentonite types, including two Na-activated bentonites, an activated Na bentonite with specifically adsorbed silica, and an active Na-Ca bentonite, exhibited differential affinity towards different PR proteins. The results obtained could be used in developing wine fining protocols which combine treatments with complementary affinity for adsorption and removal of PR proteins, and in this way achieve greater efficiency of bentonite fining by reducing its total dose, which is of significant interest to the wine industry.

## 1. Introduction

Developed protein haze or sediment in bottled white wine is considered a serious quality defect: such wines are perceived by consumers as faulty and are not marketable [1]. Protein haze in wine originates mainly from denaturation and aggregation of relatively small quantities (from 10 to several hundreds of mg/L) of the so-called pathogenesis-related (PR) grape (*Vitis vinifera* L.) proteins [2]. The PR proteins typically have lower molecular mass (15–35 kDa) and lower isoelectric point (pI), and mainly pertain to the families of thaumatin-like proteins (TLPs, PR-5 family) and chitinases (PR-3 family) [3], although a minor involvement of β-1,3-glucanase (PR-2) and ripening-related proteins in the formation of protein haze was also established [4,5]. Chitinases were recognized as primary causative agents of wine protein haze due to lower melting temperature [6], shorter half-life, and irreversible aggregation [7] in relation to other wine PR proteins. Therefore, protein instability and potential haziness in a particular wine depend not only on the total concentration of proteins, but also on their composition [4].

The current practice which has been universally employed to prevent protein haze in wine over the past 85 years is to remove proteins before bottling by adsorption onto bentonite [8]. Bentonite is a natural clay-based mineral belonging to the group of montmorillonites (aluminum hydrate silicates) with the ability to swell in water and significantly increase its size by becoming gel-type suspension [9]. From the morphological viewpoint, it has a lamellar structure alternated with exchangeable cations and hydration water. The nature of cations, prevalently Na^+^ or Ca^2+^, strongly influences some key properties of bentonite, such as, for instance, available surface and ion exchange capacity [9]. Electronegative lamellas of bentonite can interact with the protein molecules positively charged at wine pH and precipitate them. Although effective, the use of bentonite in winemaking has several drawbacks. Because of its notable swelling and poor settling properties, 3–10% of wine volume is lost during winemaking together with bentonite sediment [10], while because of its non-selectiveness, bentonite partially removes aromas, phenols, and other compounds, resulting in wines with reduced sensory quality [9,11,12,13,14,15]. Additional processing steps and long settling times delay wine marketing, while disposal of bentonite waste requires high labor input and additional costs [16,17]. Consequently, the efforts in the field have long been and still are focused on finding effective alternatives or protocols with reduced bentonite requirements. Despite being successful in the removal of PR proteins from wine, bentonite-free solutions proposed up to date, such as flash pasteurization with protease/proctase [18,19], adsorption of proteins onto ZrO_2_ [3,20,21,22], carrageenan [1,23], and, most recently, magnetic nanoparticles [24] and zeolites [25], are still at the research level, not yet implemented, and/or not allowed by European Union (EU) legislation. Among the strategies which include bentonite, in-line bentonite dosing exhibited promising results [26], but due to relatively expensive equipment and lack of practicality it is still not widely used. Another promising approach alternative to standard fining after fermentation is fining with bentonite during fermentation. This practice is relatively easily applicable, is in conformity with the current regulations, and was demonstrated to have the ability to reduce the required bentonite dose and improve wine quality [14,27,28,29]. However, its potential has not been thoroughly exploited. 

There is clear evidence that not all wine PR and other proteins, even from the same family, are adsorbed by bentonite in the same manner and proportion, and that a degree of selectivity exists. In a previous investigation, β-glucanases were found to have the strongest affinity towards bentonite and the lowest dosage was sufficient for their complete removal. Chitinases, invertases, and a fraction of TLPs were removed by a particular dose, while the rest of the TLPs (ca. 30%) were characterized by a rather weak affinity to bentonite and remained in the fined wine even after a treatment with the highest dose [5]. Jaeckels et al. [30] also observed varying adsorption behavior for different TLP isoforms, ranging from no removal to 98% reduction, and related this to the differences in protein surface hydrophobicity. Monteiro et al. [31] hypothesized that the differences in hydrophobicity of different fractions of the same protein family derive from the so-called structural micro-heterogeneities, which are a consequence of limited random proteolysis of their common precursors during winemaking. Previous findings suggest that, although not dominant in wine, proteins other than TLPs and chitinases compete with them in reaching bentonite sheets and occupying adsorption sites on its surface [5,9,27], implying that not only PR proteins should be taken into account when evaluating bentonite selectivity and efficiency. 

Differential affinity of bentonite towards different PR proteins as a function of the moment of its addition and bentonite type was hinted at in particular previous studies. Salazar et al. [17] observed that type of bentonite and the moment of its application greatly affect the extent of protein removal, the group of proteins removed, as well as the amount of lees produced and the final aromatic profile of produced wine. Lambri et al. [9] demonstrated that different types of bentonite show differential affinity towards various proteins and volatile aroma compounds. In our previous study it was shown that when added later in fermentation the efficacy of sodium-activated bentonite was higher than when added earlier. The reduction of its total dose up to 21% obtained with respect to standard fining after fermentation [14] confirmed the affinity of PR proteins towards bentonite was strongly affected by the moment of addition. Enological tannins are widely accepted as additives in modern winemaking and commercial suppliers promote their use for color enhancement, oxidative protection, and flavor and mouth-feel improvements [32,33,34]. Tannins are also well known for their interactions with proteins [35,36,37,38] and some producers declare this property of theirs on the label. However, to our knowledge, the application of commercial enological tannins as adjuvants in fining with bentonite has not been investigated up to date. 

Despite promising indications, the preferential adsorptivity of various wine proteins onto bentonite and other enological agents has not been exploited sufficiently to improve bentonite fining practice. The aim of this study was to investigate the differential affinity of various proteins towards bentonite during fermentation of grape must depending on the moment of bentonite addition, co-addition of commercial tannins, and the type of bentonite applied, at the same time monitoring the effects of these treatments on the required total bentonite dose. It was believed that the results obtained could be used in developing wine fining protocols which combine treatments with complementary affinity for adsorption and removal of PR proteins, and in this way improve the procedure by achieving greater efficiency of bentonite fining and reducing its total dose.

## 2. Materials and Methods 

### 2.1. Winemaking and Bentonite Treatments

The study included three experiments, each conducted in a separate year during the period of 2015–2017. Experiments were performed with Malvazija istarska (*Vitis vinifera* L.), the most widespread and economically important native white grape cultivar in Croatia, cultivated also in Slovenia and Italy. The grapes were harvested from the experimental vineyard of the Institute of Agriculture and Tourism (Poreč, Istria, Croatia) and were immediately destemmed, crushed, mashed, and pressed using a closed-type pneumatic press of 500 L capacity (Letina Inox d.o.o., Čakovec, Croatia) with pressure not exceeding 1 bar. The obtained juice was cold settled with the aid of Endozym Rapid pectolytic enzymes at 2 g/hL (AEB s.p.a., Brescia, Italy) for 36 h at 12 °C [14]. 

During more than 20 years of practical experience with Malvazija istarska wine protein stabilization it was observed that the doses required for complete stabilization have been lower than 150 g/L in extremely rare occasions. The arbitrarily chosen dose of 100 g/hL of granular sodium-activated bentonite (GSAB) CX Bentonite Special Grain (Corimpex, Villesse, GO, Italy) was applied in fermentation in all three experiments, since it was considered suitable for achieving partial protein stabilization during fermentation and avoiding overfining, which allowed the comparison of treatments afterwards [14].

#### 2.1.1. Experiment 1: Effect of the Moment of Bentonite Addition

The wines obtained in this experiment were used in our previous research [14]. The grapes were harvested manually on 15 September 2015. Total acidity was adjusted with the addition of 1 g/L of tartaric acid. The clear juice was homogenized and divided into 15 equal portions prepared for fermentation in stainless steel tanks of 80 L capacity. Juices were inoculated with selected yeasts *Saccharomyces cerevisiae* Lalvin QA 23 (Lallemand SA, Montreal, Canada) at 20 g/hL, rehydrated with Go-Ferm Protect Evolution (Lallemand) at 30 g/hL. Yeast supplements (25 g/L of Fermaid E, Lallemand) were added at the 2nd and 5th day of fermentation. Initial sugar concentration was 230 g/L. Fermentation was performed at 17 °C and lasted for 13 days (reducing sugars <2 g/L) [14].

GSAB was used for protein stabilization. Five treatments were set based on the moment of GSAB addition, in triplicates: (i) control (code: CO) received no GSAB, while the same GSAB dose of 100 g/hL was added in (ii) the clear juice (code: JU), (iii) at the beginning (code: BE, reducing sugars at 170–180 g/L), (iv) in the middle (code: MD, sugars at 90–100 g/L), and (v) near the end of fermentation (code: EN, sugars at 40–50 g/L) [14]. 

#### 2.1.2. Experiment 2: Effect of Co-Addition of Enological Tannins

The grapes were harvested manually on 13 September 2016. Total acidity was adjusted with the addition of 1.5 g/L of tartaric acid. The clear juice was homogenized and divided into 12 equal portions prepared for fermentation in stainless steel tanks of 80 L capacity. Juices were inoculated with selected yeasts *Saccharomyces cerevisiae* Lalvin QA 23 (Lallemand SA, Montreal, QC, Canada) at 20 g/hL, rehydrated with Go-Ferm Protect Evolution (Lallemand) at 30 g/hL. Yeast supplements (20 g/L of Fermaid E, Lallemand) were added at the 6th and 13th day of fermentation. Initial sugar concentration was 250 g/L. Fermentation was performed at 15 °C and lasted for 21–23 days.

GSAB was used for protein stabilization, while commercial enological tannin (ET) preparation Tannino Etere was supplied by Enologica Vason S.p.A. (Verona, Italy). Four treatments were set based on the moments of GSAB and ET addition, respectively, in triplicates: (i) control (code: CO) received neither GSAB nor ET, while the following treatments received (ii) 95 g/hL of GSAB near the end of fermentation (code: GSAB), (iii) 25 g/hL of ET divided in three portions on the 1st (10 g/hL), 7th (5 g/hL), and 15–17th (10 g/hL) day of fermentation (code: ET), and (iv) 95 g/hL of GSAB near the end of fermentation (sugars at 45–55 g/L) + 25 g/hL of ET divided in three portions on the 1st (10 g/hL), 7th (5 g/hL), and 15–17th (10 g/hL) day of fermentation (code: GSAB + ET). In the GSAB + ET treatment, the GSAB dose and the third dose of ET were added at the same fermentation stage but 24 h one after the other to avoid their direct interference.

#### 2.1.3. Experiment 3: Effect of Bentonite Type

The grapes were harvested manually on 6 September 2017. Total acidity was adjusted with the addition of 1.8 g/L of tartaric acid. The clear juice was homogenized and divided into 15 equal portions prepared for fermentation in stainless steel tanks of 80 L capacity. OptiMUM white (Lallemand) was added to juices at 30 g/hL. Juices were inoculated with selected yeasts *Saccharomyces cerevisiae* Lalvin QA 23 (Lallemand SA, Montreal, Canada) at 30 g/hL. Yeast supplements (20 g/L of Fermaid E, Lallemand) were added at the 4th and 11th days of fermentation. Initial sugar concentration was 205 g/L. Fermentation was performed at 15 °C and lasted for 21–23 days.

In preliminary tests relative efficacy of four different types of bentonite was determined. The following bentonites were tested: GSAB, Pentagel (PEN; activated sodium bentonite, Perdomini-IOC S.p.A., S. Martino Buon Albergo, Italy), Mastervin Compact (MVN; activated sodium bentonite with specifically adsorbed silica and activated silica, Enologica Vason), and Siha Puranit (PUR; active sodium-calcium (Na-Ca) bentonite, Eaton, Langenlonsheim, Germany). A 5 L aliquot of fresh Malvazija istarska grape juice from the experiment was stabilized by low temperature and SO_2_, and then centrifuged and filtered. For each bentonite type the doses needed to achieve complete heat stability of the grape juice were determined to the nearest 10 g/hL by the standard heat stability test (described in more detail in paragraph 2.3.): GSAB: 300 g/hL, PEN: 300 g/hL, MVN: 450 g/hL, and PUR: 750 g/hL. Accordingly, the doses intended to be applied during fermentation were adjusted proportionally to correspond to 95 g/L of GSAB. The percentage of bentonite sediment in grape juice treated with the determined doses was measured in 500-mL graduated measuring glass cylinders after 120 h at 15 °C. Five treatments were set based on the type of bentonite, in triplicates: (i) control (CO) received no bentonite, while near the end of fermentation (sugars at 35–56 g/L) the following treatments received (ii) 95 g/hL of GSAB, (iii) 95 g/hL of PEN, (iv) 143 g/hL of MVN, and (v) 238 g/hL of PUR.

### 2.2. Post-Fermentation Practices

Post-fermentation practices were the same for all the three experiments and corresponded to those already applied in our previous research [14]. After fermentation, partially stabilized wines were racked and left to spontaneously settle for 2 months. A portion from each treatment was subjected to the analysis of PR proteins (code: AFerm), while the rest of wine was fined with additional doses of GSAB required to achieve complete protein stability, as determined by the standard heat stability test (heating at 80 °C; [27,29]). After 15 days of contact with the additional dose of GSAB, protein stable wines were subjected to the analysis of PR proteins as well (code: ProStab). The level of free SO_2_ was monitored throughout the whole process and was corrected to 25–30 mg/L after fermentation, before and after racking, and before sampling, if needed [14].

### 2.3. Protein Stability Tests

The protein stability tests applied were described in our previous research [14]. Bentonite dosage rates to achieve heat stability of wines were determined to the nearest 10 g/hL by preliminary tests, using a variety of different dosages (10–300 g/hL), followed by fine tuning to the nearest 5 g/hL. For each dosage two stability tests were applied. In the standard heat stability test, wine sample (20 mL) was filtered through a PTFE 0.45 µm syringe filter and heated at 80 °C for 2 h. Sample was then shortly cooled in tap water, placed at 4 °C for another 2 h, and then left to reach room temperature. The amount of haze produced was measured by a nephelometric turbidity meter Hanna Instruments HI 83749 (Padova, Italy). A sample was considered to be protein stable when the difference between a heated sample and an unheated control was lower than 2 nephelometry turbidity units (NTU) [27,29]. In heating with tannins stability test, a portion of tannic acid solution was added to filtered wine, which was then heated at 80 °C for 2 h, and after it reached the room temperature nephelometric turbidity measurements were performed. A sample was considered to be protein stable when NTU <5 [39]. 

### 2.4. Analysis of Pathogenesis-Related (PR) Proteins by Reversed-Phase High-Performance Liquid Chromatography (RP-HPLC)

Analysis of PR proteins was carried out by reversed-phase high-performance liquid chromatography (RP-HPLC) according to the method reported by Marangon et al. [16] and modified by Van Sluyter et al. [40], using an Agilent Infinity 1260 system (Agilent Technologies, Palo Alto, CA, USA) equipped with a G1311B quaternary pump, a G1329B autosampler, a G1316A column oven, and a G4212B DAD detector. Samples were filtered through 0.45 μm PTFE filters, and 100 μL were injected onto a C8 column (4.6 × 250 mm, particle size 5 μm, Vydac 208TP54) fitted with a C8 guard column kit (4.6 × 5 mm, particle size 5 μm, Vydac 208GK54). The following gradient system was used with 0.1% (*v*/*v*) trifluoroacetic acid (TFA) in 80% acetonitrile (solvent A) and 0.1% TFA in 8% acetonitrile (solvent B): 0 min, 17% A; 7 min, 49% A, 15 min, 57% A; 16 min, 65% A; 30 min, 81% A; 35 min, 81% A; 41 min, 17% A; 51 min, 17% A. The flow was set at 1 mL/min at ambient temperature. Ultraviolet–visible (UV–Vis) detection wavelength was 210 nm, and the identity of PR proteins was assigned by comparison with the retention times reported in literature [3,16,40]: peaks with a retention time between 9 and 12 min were assigned to the TL protein classes (TL), whereas peaks eluted from 18.5 and 24.5 min were assumed to be chitinases (CHI). Semi-quantitative analysis was carried out and the concentrations of PR proteins were calculated using a calibration curve of thaumatin from *Thaumatococcus daniellii* (Sigma, St. Louis, MI, USA) assuming a relative response factor equal to one.

### 2.5. Analysis of Proteins by Size Exclusion High-Performance Liquid Chromatography (SE-HPLC)

The profiles of Malvazija istarska wine proteins based on their molecular weight (MW) were obtained using size exclusion high-performance liquid chromatography (SE-HPLC) according to the method reported by Pashova et al. [41]. Analysis was carried out using the same HPLC instrument as described above. Samples were filtered through 0.45 μm PTFE filters, and 20 μL were injected onto a TSK-Gel G2000SW column (7.5 × 300 mm, particle size 10 μm, TosoHaas GmbH, Stuttgart, Germany) with a SW-type guard column SW (7.5 × 75 mm, particle size 10 μm, TosoHaas GmbH). A 0.2 M phosphate buffer containing 0.1 M sodium chloride (Sigma, USA) was used as eluent. The phosphate buffer was obtained by mixing dibasic sodium phosphate (Sigma, USA) and monobasic sodium phosphate (Sigma, USA), and pH was adjusted to 7.0. The flow was set at 0.5 mL/min at 25 °C. UV–Vis detection wavelength was 210 nm, and the molecular weights of PR and other proteins were tentatively determined by interpolation and extrapolation based on the retention times of molecular weight standards: bovine serum albumin (BSA, MW 67 kDa, Sigma, USA), chicken egg albumin (MW 45 kDa, Sigma, USA), and lysozyme (MW 14.5 kDa, Sigma, USA). Semi-quantitative analysis was carried out and the concentrations of PR and other proteins were calculated using a calibration curve of BSA assuming a relative response factor equal to one.

### 2.6. Statistical Analysis

One-way analysis of variance (ANOVA) and Fischer’s least significant difference (LSD) test were used to compare the means (*n* = 3) at the level of significance of *p* < 0.05. Statistical data elaboration was performed with Statistica v. 13.2 software (Stat-Soft Inc., Tulsa, OK, USA).

## 3. Results and Discussion

### 3.1. Experiment 1: Effect of the Moment of Bentonite Addition

**Figure 1:**
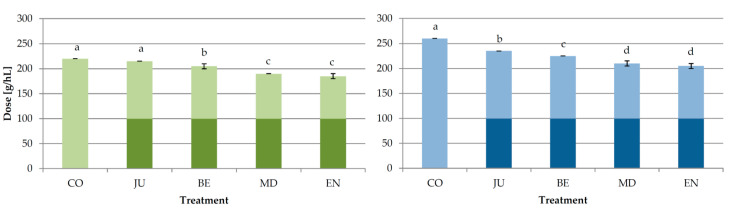
Doses of bentonite required for protein stabilization of wines in different treatments of Experiment 1 determined by heating stability test (green bars) and heating with tannins test (blue bars), respectively [14]. CO—control wine without bentonite in fermentation, JU—initial granular sodium-activated bentonite dose added into clear juice, BE—initial granular sodium-activated bentonite dose added at the beginning of fermentation, MD—initial granular sodium-activated bentonite dose added in the middle of fermentation, EN—initial granular sodium-activated bentonite dose added near the end of fermentation. The wines were treated by additional granular sodium-activated bentonite doses after fermentation to achieve total protein stability. Dark color segments of the bars represent initial doses, and light color segments represent additional doses. Different lowercase letters above bars represent statistically significant differences among treatments with respect to total bentonite dose required, at *p* < 0.05 obtained by one-way analysis of variance (ANOVA) and Fischer’s least significant difference (LSD) test.

**Figure 2:**
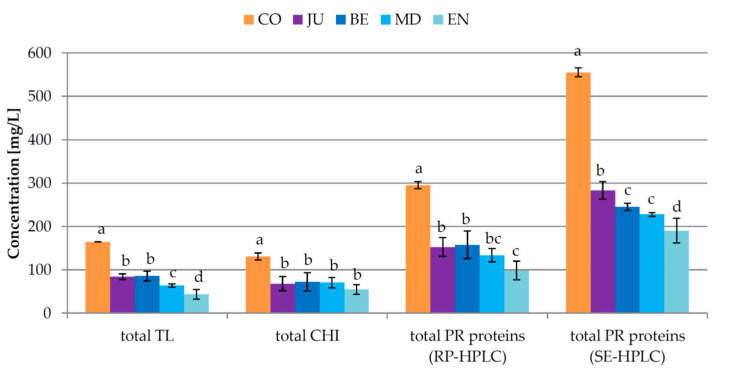
Total concentrations of pathogenesis-related (PR) proteins in Malvazija istarska wines (mean ± standard deviation; *n* = 3) obtained after partial fining with bentonite at different points of fermentation. CO—control wine without bentonite in fermentation, JU—initial granular sodium-activated bentonite dose added into clear juice, BE—initial granular sodium-activated bentonite dose added at the beginning of fermentation, MD—initial granular sodium-activated bentonite dose added in the middle of fermentation, EN—initial granular sodium-activated bentonite dose added near the end of fermentation. TL—thaumatin-like proteins, CHI—chitinases, RP-HPLC—reverse phase high-performance liquid chromatography, SE-HPLC—size exclusion high-performance liquid chromatography. Different lowercase letters above bars represent statistically significant differences among treatments with respect to total bentonite dose required, at *p* < 0.05 obtained by one-way ANOVA and LSD test.

Initial (added during fermentation), additional (added after fermentation), and total (initial + additional) doses of GSAB applied in different phases of fermentation in order to achieve protein stable Malvazija istarska wines were determined previously [14] and are reported in Figure 1. All the treatments involving bentonite addition during fermentation significantly reduced the total amount required in relation to standard fining after fermentation in CO treatment according to heating with tannins stability test. The most effective were the treatments MD and EN with the reduction with respect to CO of approximately 14% and 16% (heating test) or 19% and 21% (heating with tannins test), respectively [14]. In the produced wines, four TL proteins and two chitinases were tentatively identified by RP-HPLC, while seven fragments of different molecular weight, among which five with weights commonly considered to correspond to PR protein species (from 20 to 35 kDa; [41,42,43]), were tentatively identified by SE-HPLC. All the treatments significantly reduced the concentrations of all the identified proteins compared to CO wine (Table 1, Figure 2 and Figure S1). The amount of additional bentonite needed for complete fining of the obtained wines was in a positive correlation with the concentrations of particular as well as total residual PR proteins determined by both HPLC techniques after fermentation, and therefore MD and especially EN wines contained the lowest concentrations. 

Although in EN wine a clear tendency towards decrease in concentration was noted for all the PR proteins identified by RP-HPLC, statistically lower concentrations with respect to the other treatments with bentonite were observed only for TL2 and TL4, as well as total TL proteins. As for the SE-HPLC results, all the treatments with bentonite lowered the concentration of P93, P67, PR32, PR25, and total PR proteins (Table 1, Figure 2). EN treatment was the most effective in the removal of the rest of the PR proteins (PR23–20), followed by MD in most cases, which mostly corresponded to the RP-HPLC results for TL2 and TL4. Since it was estimated previously that the theoretical molecular weight of TLPs is around 23 kDa, while the weight of chitinases is slightly higher, around 27 kDa [42] or 35 kDa [43], respectively, it is possible that the PR proteins tentatively identified in this work with the estimated molecular weight between 20 to 23 kDa at least partly corresponded to the TL proteins identified by RP-HPLC.

It is probable that the overall must or wine matrix composition, which certainly varied depending on the moment of bentonite addition, had a significant influence on the efficacy of fining and removal of PR proteins during fermentation. It was shown earlier that various wine components and parameters, such as different ions [12], tannins [44,45], polysaccharides [46], and pH value [12] may significantly affect PR protein stability, so it is possible that the matrix affected their affinity towards bentonite as well. Among other components, ethanol and reducing sugars varied most significantly among the treatments with respect to the moment of bentonite addition. It was demonstrated previously that a particular bentonite had a maximum chicken egg albumin (ovalbumin) adsorption capacity in wine model solution at around 11 vol.% of ethanol [47]. This corresponded to the alcoholic strength of wine at the moment of the bentonite addition in EN treatment in this work (data not shown). It was hypothesized that ethanol increases the swelling and adsorption capacity of bentonite by displacing smaller water molecules in bentonite layers [48]. It was shown previously that the effect of ethanol may vary depending on the size of protein molecule. The adsorption of smaller proteins by bentonite was enhanced by increasing ethanol levels up to 10 vol.% for bovine serum albumin (BSA) and up to 12 vol.% for lysozyme, while the volume fraction of ethanol had no significant effect on the adsorption of ovalbumin [49]. PR fractions (20–35 kDa, [41,42,43]) are generally among the smaller proteins in wine, so it is reasonable to assume that ethanol has the ability to separate bentonite layers enough to enhance their adsorption to a certain degree. It is possible that the varying ethanol content was indeed among the main reasons why the degree of the reduction of PR protein concentrations and bentonite dose depended on the time of addition. It is possible that particular TL proteins for which a significant decrease in MD and EN wines was noted, namely TL2 and TL4, or PR23, PR 22, and PR20 (Table 1), respectively, were the most susceptible to such interaction because of their matching macromolecular shape and size. The results obtained in this study corresponded to those obtained by other groups who also found additions later in fermentation to be the most effective [27,29]. 

Total must protein amount decreases during the course of fermentation [29]. It is possible that when added earlier, although longer in contact, a part of bentonite was spent by adsorbing particular non- or less pathogenic proteins as well as other solid particles and macromolecules present in higher concentration at that stage of fermentation, and was therefore less effective in capturing the PR ones in JU and BE than in MD and EN treatments, respectively. Although apparently not so significant when considered in relation to the total protein concentration, higher efficiency of the removal of P93 in JU than in EN treatment corroborated this assumption. In an earlier report it was hypothesized that limited proteolysis of common protein precursors from grapes generates a large number of structurally related but different proteins during winemaking [31], which was corroborated by other findings [5]. It is possible that during fermentation subtle micro-heterogeneities in protein molecular structure have developed which enhanced the affinity of particular PR proteins, namely TL2 and TL4 or PR23–20 proteins, towards bentonite in the later phases of fermentation. 

The general preferential affinity of various PR proteins towards bentonite during fermentation turned out to be rather different. Considering the RP-HPLC results, TL1 and TL4 concentrations were reduced the most, from ca. 48% to 97% depending on the treatment, while TL3 was the most resistant with the reduction from just 14% to 32% (Table S1). The weakest affinity of TL3 towards bentonite was confirmed after the additional fining after fermentation, since it had the lowest removal rate and was represented by the highest proportion in the residual PR protein fraction. Such results were in accordance with the findings from Sauvage et al. [5] and Jaeckels et al. [30] who also observed a dual response of TLPs, with a fraction successfully removed by a particular bentonite dose, and the rest of TLPs remaining in wine even after treatments with rather high doses. In this work, on average, TLPs exhibited slightly higher affinity towards bentonite than chitinases during fermentation (Table S1), while after additional fining the opposite tendency was observed. The SE-HPLC analysis showed that PR32, found in a very low concentration in CO wine, was removed completely during fermentation by all the investigated treatments. Proteins with lower molecular weight, PR20 and PR22, exhibited stronger affinity to bentonite than the other tentatively identified proteins. 

### 3.2. Experiment 2: Effect of Co-Addition of Enological Tannins

**Figure 3:**
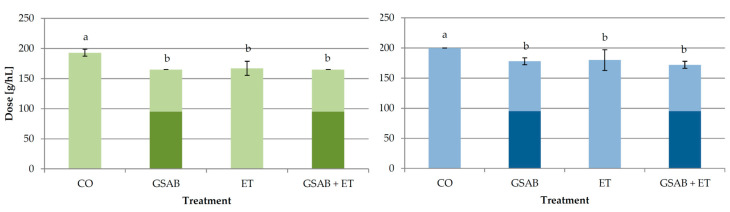
Doses of bentonite required for protein stabilization of wines in different treatments of Experiment 2, determined by heating stability test (green bars) and heating with tannins test (blue bars), respectively. CO—control wine without bentonite or commercial enological tannin preparation added during fermentation, GSAB—initial dose of granular sodium-activated bentonite added near the end of fermentation, ET—commercial enological tannin preparation added during fermentation, GSAB + ET—initial dose of granular sodium-activated bentonite added near the end of fermentation and commercial enological tannin preparation added during fermentation. The wines were treated by additional granular sodium-activated bentonite doses after fermentation to achieve total protein stability. Dark color segments of the bars represent initial doses, and light color segments represent additional doses. Different lowercase letters above bars represent statistically significant differences among treatments with respect to total bentonite dose required, at *p* < 0.05 obtained by one-way ANOVA and LSD test.

The doses of GSAB (initial, additional, and total) applied during fermentation with or without ET in order to achieve protein stable Malvazija istarska wines are shown in Figure 3. As in Experiment 1, GSAB treatment (addition of bentonite near the end of fermentation) significantly reduced the total dose required in relation to control CO wine. Interestingly, ET treatment reduced the total dose to the same extent. Although GSAB + ET treatment reduced the total dose needed, the effects of GSAB and ET did not add up when combined.

**Figure 4:**
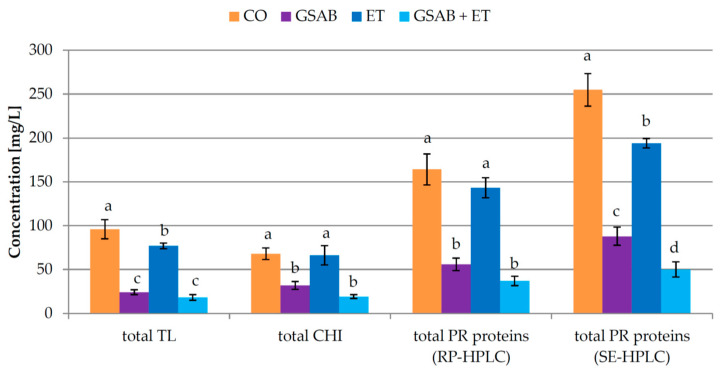
Total concentrations of pathogenesis-related (PR) proteins in Malvazija istarska wines (mean ± standard deviation; *n* = 3) obtained after partial fining with bentonite and/or the addition of commercial enological tannin preparation during fermentation. CO—control wine without bentonite or commercial enological tannin preparation added during fermentation, GSAB—initial dose of granular sodium-activated bentonite added near the end of fermentation, ET—commercial enological tannin preparation added during fermentation, GSAB + ET—initial dose of granular sodium-activated bentonite added near the end of fermentation and commercial enological tannin preparation added during fermentation. TL—thaumatin-like proteins, CHI—chitinases, RP-HPLC—reverse phase high-performance liquid chromatography, SE-HPLC—size exclusion high-performance liquid chromatography. Different lowercase letters above bars represent statistically significant differences among treatments with respect to total bentonite dose required, at *p* < 0.05 obtained by one-way ANOVA and LSD test.

The addition of bentonite in GSAB treatment reduced the concentration of all the investigated PR proteins in relation to CO after fermentation (Table 2, Figure 4 and Figure S2). Similar as in Experiment 1, the most affected were TL4 and TL1, followed by TL2 and chitinases, while TL3 was again the most resistant (Table S2). Also, PR22 and PR20 were reduced to a greater extent than the other PR proteins, except PR32 whose already low concentration was reduced to zero after fermentation. ET treatment during fermentation reduced the concentrations of TLPs to a certain extent, but did not affect chitinases notably. From the SE-HPLC perspective, ET reduced the concentrations of all the protein weights, but to a much lesser degree than GSAB. 

It is possible that the enological tannins added during ET treatment interacted with particular proteins to a certain degree and formed precipitates which were removed by racking or remained soluble. It was previously reported that tannin-protein interactions are influenced by the characteristics of a protein, including its size, amino acid composition, pI, and extent of post-translational modification. Tannin-protein complexes are normally established by hydrogen bonds and hydrophobic interactions, so the affinity of a protein towards tannin phenolic hydroxyl groups presumably depended on the number of the available peptide bonds, whose carbonyl oxygen serves as the main hydrogen bond acceptor [35]. Soluble complexes are formed when protein is present in excess, in which case each protein molecule is bound by only a few tannin ligands and the complex is not hydrophobic enough to precipitate [35]. In a medium abundant in tannins, as in the case of ET and GSAB + ET treatments in this study, the possibility of the formation of higher concentrations of hydrophobic and precipitable protein-tannin complexes was certainly higher. GSAB and ET showed a combined effect in reducing CHI1 concentration, while the concentrations of the majority of other PR proteins determined by RP-HPLC which remained in the wines after fermentation were also lower than in GSAB wine, although without a significant difference. A synergistic effect of GSAB + ET was observed in removing PR25, PR23, and PR22 proteins. Besides the assumed removal of insoluble complexes by precipitation, it is possible that particular non-precipitable complexes of tannins with chitinases and other PR proteins were more susceptible to adsorption on bentonite in GSAB + EN treatment than proteins alone. However, the combined effect of GSAB + ET was obviously not capable to decrease the total bentonite dose required in relation to the treatment with GSAB alone (Figure 3). It is probable that the soluble complexes formed which remained in wine were also instable, contributed to the measured turbidity, and required a particular bentonite dose to be removed.

When comparing the percentages of the removal of PR proteins, higher removal rate of all the investigated PR proteins, as well as total proteins in Experiment 2 than in Experiment 1 (Table 1 and Table 2, Figure 2 and Figure 4) was in line with previous findings showing wine with less initial protein exhibiting higher percentage of protein removal, i.e., increased adsorption of proteins on bentonite at low solute concentration [48,49,50].

### 3.3. Experiment 3: Effect of Bentonite Type

**Figure 5:**
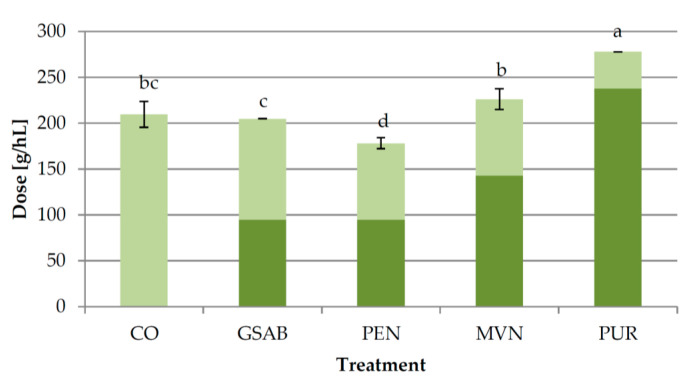
Doses of bentonite required for protein stabilization of wines in different treatments of Experiment 3 determined by heating stability test. CO—control wine without bentonite in fermentation, GSAB—initial dose of granular sodium-activated bentonite CX Special Grain added near the end of fermentation, PEN—initial dose of sodium-activated bentonite Pentagel added near the end of fermentation, MVN—initial dose of activated sodium bentonite with specifically adsorbed silica and activated silica Mastervin Compact added near the end of fermentation, PUR—initial dose of active sodium-calcium bentonite Siha Puranit added near the end of fermentation. The wines were treated by additional granular sodium-activated CX Special Grain bentonite doses after fermentation to achieve total protein stability. Dark color segments of the bars represent initial doses, and light color segments represent additional doses. Different lowercase letters above bars represent statistically significant differences among treatments with respect to total doses applied, both at *p* < 0.05 obtained by one-way ANOVA and LSD test.

The doses of different types of bentonite with the same efficiency in achieving protein stability of grape juice were determined in a preliminary experiment (Section 2.1.3). However, their efficiency during fermentation was not the same. Lower additional bentonite doses were needed to achieve complete protein stability of wines in PEN, MVN, and PUR treatments than in GSAB treatment (Figure 5). This clearly showed that the pre-determined doses of PEN (95 g/hL), MVN (143 g/hL), and PUR (238 g/hL) were more efficient than the GSAB dose (95 g/hL) when applied in fermentation. It is probable that the differences in the composition of grape juice and must in the later stages of fermentation, as well as the changes in the content and composition of PR proteins during fermentation, were the main causes of the observed differences. Based on the newly established ratios, the average doses with the efficiency for lowering bentonite dose equivalent to 95 g/hL of GSAB were estimated as ca. 74 g/hL of PEN as the most efficient, followed by 112 g/hL of MVN and 138 g/hL of PUR. (Example of calculation: after the initial application of 95 g/hL of GSAB applied in fermentation, the average additional dose of GSAB applied after fermentation to achieve total protein stability was 110 g/L, meaning the average total dose was 205 g/hL. After the application of the same dose of PEN (95 g/hL) in fermentation, the average additional dose of GSAB applied after fermentation to achieve total protein stability of PEN treatment wine was 83 g/hL, meaning 95 g/hL of PEN was about as efficient as 122 g/hl of GSAB (205 – 83 = 122 g/hL). According to that, PEN was 1.28 times more efficient than GSAB (122/95), so the dose of PEN with the same efficiency as 95 g/hL of GSAB was calculated by dividing the dose of GSAB 95 g/hL by 1.28, which is 74 g/L.) This further implied that the applied doses of PEN, MVN, and PUR were 1.28, 1.28, and 1.72 times more efficient than the GSAB dose applied in fermentation, respectively. 

Another aspect which is important when comparing fining with different bentonite types from the point of view of economic viability is the amount of bentonite sediment produced. After the application of the preliminarily determined dosages (Figure 5) the ratios of the sediment volumes with respect to the sediment formed in GSAB treatment, to which a reference value of 1.0 was arbitrarily assigned, were as follows: PEN 1.4, MVN 0.5, and PUR 0.13 (Figure S3). The lowest volume of sediment produced by the active Na-Ca bentonite in PUR treatment despite its highest dose corroborated the practical knowledge about the lower swelling capacity of bentonites with a high proportion of calcium. Although not measured, it is very reasonable to assume that PUR treatment had the highest wine yield. The efficacy of silica as a coadjuvant to obtain more compact lees was confirmed in MVN treatment. The large differences between the treatments in the volume of sediment obtained confirmed that several factors need to be considered when planning the fining procedure, of which the efficiency of bentonite and the amount of sediment are among the most important. It is worth mentioning that the addition of bentonite late in fermentation in GSAB treatment did not result with a reduced total bentonite dose as in the previous two experiments (Section 3.1 and Section 3.2). However, the doses of the other types of bentonite used did (Figure 5). Apart from the possible differences between the wine matrices due to the effect of harvest year, which was shown to have an effect in previous studies [51], lower amount of ethanol formed at the moment of dosing during fermentation in Experiment 3 than in Experiments 1 and 2 (data not shown) could have also had an effect. 

**Figure 6:**
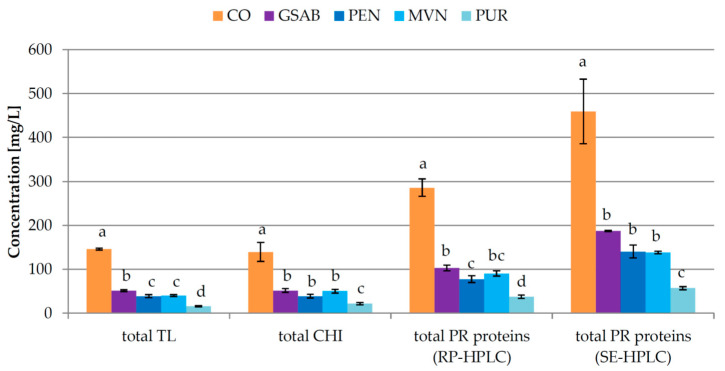
Total concentrations of pathogenesis-related (PR) proteins in Malvazija istarska wines (mean ± standard deviation; *n* = 3) obtained after partial fining with different types of bentonite in fermentation. CO—control wine without bentonite in fermentation, GSAB—initial dose (95 g/hL) of granular sodium-activated bentonite CX Special Grain added near the end of fermentation, PEN—initial dose (95 g/hL) of sodium-activated bentonite Pentagel added near the end of fermentation, MVN—initial dose (143 g/hL) of activated sodium bentonite with specifically adsorbed silica and activated silica Mastervin Compact added near the end of fermentation, PUR—initial dose (238 g/hL) of active sodium-calcium bentonite Siha Puranit bentonite added near the end of fermentation. TL—thaumatin-like proteins, CHI—chitinases, RP-HPLC—reverse phase high-performance liquid chromatography, SE-HPLC—size exclusion high-performance liquid chromatography. Different lowercase letters above bars represent statistically significant differences among treatments with respect to total bentonite dose required, at *p* < 0.05 obtained by one-way ANOVA and LSD test.

As expected, the concentrations of all the investigated PR proteins were significantly reduced by each of the bentonites applied in fermentation with respect to CO wine (Table 3, Figure 6 and Figure S4). Similar as in Experiments 1 and 2, the percentage of the removal by GSAB during fermentation was the highest for TL4, slightly lower for TL1 and chitinases followed by TL2, and the lowest for TL3 (Table S3). The other bentonites showed similar tendencies, although in different magnitudes. From the SE-HPLC perspective, P92 was reduced to a similar extent by all the bentonite types, only PUR removed a portion of P67, while the concentration of PR32 was reduced to zero by all the treatments. 

For the majority of PR proteins, the applied doses of the other bentonite types were more efficient than GSAB (Table 3, Figure 6). This was especially evident for TL4 and TL1, as well as PR23–20, while TL3, presumably the most resilient to bentonite adsorption among them, was most efficiently removed by GSAB. As for chitinases, the efficiency of the applied dosages of GSAB, PEN, and MVN did not statistically differ, although PEN showed a tendency to reduce chitinases more. PUR dosage was the most efficient in the removal of all the investigated PR proteins as revealed by both RP-HPLC and SE-HPLC analysis. Since the four bentonites were applied in different doses which had different relative efficiencies in improving the protein stability of the investigated wines (Figure 5), it was hard to estimate precisely their relative affinities towards various PR proteins. GSAB and PEN were the only bentonites added at the same dose and it can be stated with certainty that PEN was generally more efficient in the removal of TL1, TL4, total TL, and total RP-HPLC proteins, as well as PR23–20 proteins. It was roughly estimated that the majority of PR proteins determined by RP-HPLC, except TL4, showed a greater affinity towards GSAB and PEN than to MVN, which, although applied in a larger dose, did not remove proportionally more PR proteins. Differential affinity of PR proteins towards various bentonites was also observed in previous studies. Salazar et al. [17] noted that sodium bentonite added in grape juice before fermentation effectively targeted PR proteins, while sodium activated bentonite seemed to be generally less effective. However, the later produced ca. 40% lower amount of sediment. Lambri et al. [9] observed differences among the efficacies of three sodium activated bentonites. The authors related them to the variability in the physico-chemical characteristics of the bentonites, namely their cation composition, surface charge density, the external specific surface area, and swelling capacity. It is probable that the same variables strongly affected the differences in the efficacy of bentonites investigated in this study.

In Experiment 3 a deviation from the tendency noted in Experiments 1 and 2 was observed, with TL proteins and chitinases removed in similar percentages by the application of the majority of the studied bentonite types (Table S3). When comparing Experiments 2 and 3 with respect to chitinases and total PR protein concentration, a higher percentage of removal was noted in Experiment 3 despite the almost doubly higher initial concentration than in Experiment 2 (Table 2 and Table 3, Figure 4 and Figure 6). This was also an exception from the previously noted patterns with increased adsorption of proteins on bentonite at lower protein concentration [48,49,50]. Such discrepancies once again highlighted the possible key impact of the harvest year and different starting physico-chemical composition of grape juice on bentonite fining efficiency, which has to be considered in future studies.

## 4. Conclusions

The results of this study showed that the moment of bentonite addition, co-addition of tannins, and bentonite type significantly affect the affinity of PR proteins towards bentonite during fermentation. Different proportions of removal not only among various protein families, but also among proteins from the same family were observed depending on the investigated factors, confirming once again the variety and the complexity of bentonite–protein interactions in wine. It was shown that dosing bentonite in the later phases of fermentation and the application of exogenous enological tannins may improve the efficacy of fining. The application of different types of bentonite significantly affected the PR protein content and composition, fining efficacy, and the volume of bentonite sediment. The results obtained could be used in developing wine fining protocols which combine treatments with complementary affinity for adsorption and removal of PR proteins, and in this way achieve greater efficiency of bentonite fining by reducing its total dose, which is of significant interest to the wine industry.

## Figures and Tables

**Table 1:** Concentrations of proteins in Malvazija istarska wines (mean ± standard deviation; *n* = 3; mg/L) obtained after partial fining with bentonite at different points of fermentation, and in final protein-stable wines.

Protein	Stage	Treatment				
		CO	JU	BE	MD	EN
*RP-HPLC* ^1^						
TL1	AFerm	54.45 ± 1.54 a	26.04 ± 3.80 b	28.41 ± 11.30 b	23.91 ± 3.52 b	17.45 ± 4.88 b
	ProStab	1.37 ± 0.37 a	0.74 ± 0.11 b	0.66 ± 0.05 b	0.48 ± 0.26 b	0.69 ± 0.13 b
TL2	AFerm	22.85 ± 0.19 a	15.47 ± 0.65 b	15.18 ± 1.59 b	14.8 ± 0.47 b	10.85 ± 2.36 c
	ProStab	3.24 ± 0.80 a	1.63 ± 0.57 b	1.53 ± 0.21 b	1.55 ± 0.10 b	2.41 ± 0.17 a
TL3	AFerm	19.17 ± 0.54 a	15.00 ± 0.85 ab	15.55 ± 2.60 ab	16.4 ± 0.96 ab	12.96 ± 3.14 b
	ProStab	6.91 ± 1.74 a	2.39 ± 0.94 c	2.52 ± 0.47 c	2.74 ± 0.33 c	4.22 ± 0.36 b
TL4	AFerm	67.76 ± 0.80 a	27.87 ± 3.55 b	26.49 ± 4.13 b	8.34 ± 1.46 c	2.30 ± 1.01 d
	ProStab	0.97 ± 0.63	0.72 ± 0.04	0.58 ± 0.12	0.81 ± 0.17	0.77 ± 0.07
CHI1	AFerm	73.66 ± 5.81 a	38.1 ± 9.79 b	41.74 ± 12.35 b	40.84 ± 6.80 b	32.72 ± 6.09 b
	ProStab	0.95 ± 0.01	0.64 ± 0.21	0.87 ± 0.06	0.91 ± 0.03	0.84 ± 0.07
CHI2	AFerm	57.23 ± 2.47 a	29.89 ± 6.50 b	30.31 ± 8.86 b	29.27 ± 5.26 b	21.82 ± 4.84 b
	ProStab	0.16 ± 0.03	0.18 ± 0.02	0.15 ± 0.01	0.17 ± 0.06	0.16 ± 0.02
*SE-HPLC* ^2^						
P93	AFerm	13.89 ± 0.56 a	10.19 ± 0.12 c	10.41 ± 1.08 bc	11.30 ± 1.03 bc	11.98 ± 0.66 b
	ProStab	11.00 ± 0.57	9.58 ± 0.54	9.94 ± 1.23	9.84 ± 0.98	11.22 ± 0.92
P67	AFerm	13.27 ± 1.81 a	7.38 ± 0.92 b	5.97 ± 0.98 b	7.15 ± 0.39 b	6.12 ± 1.13 b
	ProStab	0.00 ± 0.00	0.00 ± 0.00	0.00 ± 0.00	0.00 ± 0.00	0.00 ± 0.00
PR32	AFerm	4.30 ± 1.66 a	0.00 ± 0.00 b	0.00 ± 0.00 b	0.00 ± 0.00 b	0.00 ± 0.00 b
	ProStab	0.00 ± 0.00	0.00 ± 0.00	0.00 ± 0.00	0.00 ± 0.00	0.00 ± 0.00
PR25	AFerm	228.15 ± 3.64 a	119.85 ± 10.56 b	103.85 ± 1.02 b	107.91 ± 3.28 b	101.61 ± 16.13 b
	ProStab	26.20 ± 8.06 a	5.01 ± 1.97 b	5.00 ± 0.29 b	4.51 ± 1.18 b	8.62 ± 0.68 b
PR23	AFerm	90.07 ± 2.11 a	61.95 ± 5.76 b	48.47 ± 0.49 cd	52.84 ± 0.63 c	43.33 ± 6.96 d
	ProStab	9.36 ± 3.89 a	2.03 ± 1.09 b	1.58 ± 0.04 b	1.74 ± 0.49 b	4.00 ± 0.55 b
PR22	AFerm	135.15 ± 3.32 a	56.08 ± 6.87 b	52.19 ± 6.46 b	35.39 ± 2.76 c	20.41 ± 3.83 d
	ProStab	8.23 ± 3.23 a	1.97 ± 0.85 b	2.09 ± 0.03 b	1.71 ± 0.44 b	2.84 ± 0.30 b
PR20	AFerm	97.54 ± 7.11 a	45.31 ± 0.71 b	40.60 ± 1.86 b	31.42 ± 4.28 c	25.02 ± 2.00 c
	ProStab	14.81 ± 0.41 a	9.34 ± 0.32 b	10.18 ± 0.93 b	7.89 ± 1.09 c	7.48 ± 0.52 c

TL—thaumatin-like proteins, CHI—chitinases, P93–PR20—proteins with numbers denominating the estimated molecular weight in kDa, CO—control wine without bentonite in fermentation, JU—initial granular sodium-activated bentonite dose (100 g/hL) added into clear juice, BE—initial granular sodium-activated bentonite dose (100 g/hL) added at the beginning of fermentation, MD—initial granular sodium-activated bentonite dose (100 g/hL) added in the middle of fermentation, EN—initial granular sodium-activated bentonite dose (100 g/hL) added near the end of fermentation. AFerm—wines analyzed after fermentation, ProStab—wines analyzed after total protein stabilization by additional post-fermentation fining with granular sodium-activated bentonite. ^1^ Concentrations are expressed as equivalents of thaumatin from *Thaumatococcus daniellii*. ^2^ Concentrations are expressed as equivalents of bovine serum albumin. Different lowercase letters in a row represent statistically significant differences among treatments, at *p* < 0.05 obtained by one-way ANOVA and LSD test.

**Table 2:** Concentrations of proteins in Malvazija istarska wines (mean ± standard deviation; *n* = 3; mg/L) obtained after partial fining with bentonite and/or the addition of commercial enological tannin preparation during fermentation, and in final protein stable wines.

Protein	Stage	Treatment			
		CO	GSAB	ET	GSAB + ET
*RP-HPLC* ^1^					
TL1	AFerm	38.11 ± 6.14 a	8.31 ± 1.71 c	29.03 ± 3.50 b	4.00 ± 1.34 c
	ProStab	1.40 ± 1.78	1.05 ± 0.89	1.41 ± 0.94	0.29 ± 0.07
TL2	AFerm	13.87 ± 1.16 a	5.48 ± 0.41 c	11.34 ± 1.13 b	3.97 ± 0.92 c
	ProStab	1.89 ± 0.67	1.89 ± 0.48	1.66 ± 0.02	1.68 ± 0.20
TL3	AFerm	13.94 ± 0.69 a	9.61 ± 0.26 b	12.37 ± 2.06 a	9.76 ± 1.35 b
	ProStab	3.85 ± 0.97	4.90 ± 0.83	3.46 ± 0.25	4.23 ± 0.54
TL4	AFerm	30.17 ± 3.17 a	0.73 ± 0.53 c	24.16 ± 1.69 b	0.29 ± 0.19 c
	ProStab	0.70 ± 0.08	0.00 ± 0.00	1.21 ± 0.86	0.06 ± 0.10
CHI1	AFerm	36.63 ± 3.38 a	17.42 ± 2.17 b	34.66 ± 5.58 a	10.49 ± 1.27 c
	ProStab	3.49 ± 2.44	4.74 ± 2.09	3.95 ± 2.05	2.14 ± 1.00
CHI2	AFerm	31.45 ± 3.27 a	14.44 ± 2.25 b	31.62 ± 5.30 a	8.55 ± 0.94 b
	ProStab	2.80 ± 1.63	4.28 ± 1.59	3.75 ± 1.87	1.82 ± 0.76
*SE-HPLC* ^2^					
P93	AFerm	8.38 ± 0.84	8.05 ± 0.03	7.12 ± 1.11	7.26 ± 1.32
	ProStab	8.88 ± 0.74	7.86 ± 0.20	9.04 ± 0.95	8.91 ± 0.11
P67	AFerm	7.55 ± 0.63 a	4.64 ± 0.12 bc	4.85 ± 0.46 b	3.00 ± 1.59 c
	ProStab	0.90 ± 0.85	0.90 ± 0.35	0.32 ± 0.55	0.00 ± 0.00
PR32	AFerm	5.13 ± 0.42 a	0.00 ± 0.00 c	3.63 ± 0.45 b	0.00 ± 0.00 c
	ProStab	0.00 ± 0.00	0.00 ± 0.00	0.00 ± 0.00	0.00 ± 0.00
PR25	AFerm	94.65 ± 5.64 a	44.94 ± 5.49 c	71.52 ± 1.25 b	24.99 ± 2.58 d
	ProStab	11.09 ± 7.56	12.77 ± 3.45	7.22 ± 2.13	7.27 ± 1.68
PR23	AFerm	55.60 ± 6.47 a	21.51 ± 2.98 c	38.99 ± 1.78 b	10.36 ± 2.63 d
	ProStab	5.12 ± 3.79	4.39 ± 1.40	2.83 ± 1.32	2.53 ± 0.92
PR22	AFerm	41.51 ± 2.38 a	7.40 ± 1.12 c	34.47 ± 2.32 b	3.01 ± 1.12 d
	ProStab	3.33 ± 2.05 a	1.19 ± 0.50 b	0.38 ± 0.48 b	0.18 ± 0.16 b
PR20	AFerm	58.04 ± 4.45 a	14.09 ± 0.69 c	45.23 ± 2.32 b	11.67 ± 2.36 c
	ProStab	10.69 ± 1.50 a	6.84 ± 0.40 c	7.38 ± 0.56 bc	9.19 ± 1.02 ab

TL—thaumatin-like proteins, CHI—chitinases, P93–PR20—proteins with numbers denominating the estimated molecular weight in kDa, CO—control wine without bentonite or commercial enological tannin preparation added during fermentation, GSAB—initial dose (95 g/hL) of granular sodium-activated bentonite added near the end of fermentation, ET—commercial enological tannin preparation (25 g/hL divided in three portions) added during fermentation, GSAB + ET—initial dose (95 g/hL) of granular sodium-activated bentonite added near the end of fermentation and commercial enological tannin preparation (25 g/hL divided in three portions) added during fermentation. AFerm—wines analyzed after fermentation, ProStab—wines analyzed after total protein stabilization by additional post-fermentation fining with bentonite. ^1^ Concentrations are expressed as equivalents of thaumatin from *Thaumatococcus daniellii*. ^2^ Concentrations are expressed as equivalents of bovine serum albumin. Different lowercase letters in a row represent statistically significant differences among treatments, at *p* < 0.05 obtained by one-way ANOVA and LSD test.

**Table 3:** Concentrations of proteins in Malvazija istarska wines (mean ± standard deviation; *n* = 3; mg/L) obtained after partial fining with different types of bentonite in fermentation, and in final protein stable wines.

Protein	Stage	Treatment				
		CO	GSAB	PEN	MVN	PUR
*RP-HPLC* ^1^						
TL1	AFerm	66.79 ± 1.15 a	22.08 ± 1.49 b	16.88 ± 2.20 c	17.86 ± 1.24 c	3.90 ± 0.74 d
	ProStab	2.83 ± 0.57 a	2.50 ± 0.08 ab	2.33 ± 0.06 b	1.88 ± 0.17 c	0.60 ± 0.15 d
TL2	AFerm	17.13 ± 0.43 a	8.62 ± 0.55 b	8.77 ± 0.39 b	9.16 ± 0.49 b	4.46 ± 0.36 c
	ProStab	1.17 ± 0.04 b	1.11 ± 0.02 b	1.66 ± 0.25 a	1.40 ± 0.12 ab	1.15 ± 0.11 b
TL3	AFerm	13.47 ± 0.05 a	9.61 ± 0.70 c	11.67 ± 0.71 b	12.03 ± 0.58 b	6.83 ± 0.33 d
	ProStab	1.56 ± 0.13 b	1.51 ± 0.03 b	2.63 ± 0.48 a	2.30 ± 0.42 a	2.41 ± 0.07 a
TL4	AFerm	48.68 ± 0.58 a	11.01 ± 0.58 b	1.54 ± 0.95 c	1.09 ± 0.17 c	0.73 ± 0.08 c
	ProStab	1.33 ± 0.55 a	0.94 ± 0.11 a	0.14 ± 0.09 b	0.07 ± 0.02 b	0.03 ± 0.04 b
CHI1	AFerm	77.06 ± 9.40 a	28.64 ± 2.48 b	22.89 ± 2.40 b	29.44 ± 2.55 b	12.78 ± 1.37 c
	ProStab	3.88 ± 0.63	3.23 ± 0.10	3.28 ± 0.16	3.38 ± 0.30	2.96 ± 0.49
CHI2	AFerm	62.52 ± 12.46 a	23.05 ± 2.11 b	15.77 ± 1.93 bc	20.87 ± 1.73 b	8.89 ± 0.90 c
	ProStab	2.93 ± 0.77	2.55 ± 0.06	2.31 ± 0.04	2.54 ± 0.29	1.98 ± 0.42
*SE-HPLC* ^2^						
P93	AFerm	7.25 ± 0.82 a	5.80 ± 0.36 b	5.69 ± 0.82 b	5.44 ± 0.10 b	5.07 ± 0.38 b
	ProStab	6.35 ± 0.56	5.53 ± 0.51	5.31 ± 0.55	5.14 ± 0.32	4.89 ± 0.41
P67	AFerm	6.68 ± 1.07 a	6.00 ± 0.42 a	6.54 ± 0.45 a	6.83 ± 0.56 a	3.92 ± 0.25 b
	ProStab	0.74 ± 0.14	0.82 ± 0.06	1.22 ± 0.07	1.14 ± 0.25	1.24 ± 0.51
PR32	AFerm	4.04 ± 0.75 a	0.00 ± 0.00 b	0.00 ± 0.00 b	0.00 ± 0.00 b	0.00 ± 0.00 b
	ProStab	0.00 ± 0.00	0.00 ± 0.00	0.00 ± 0.00	0.00 ± 0.00	0.00 ± 0.00
PR25	AFerm	181.45 ± 43.16 a	85.81 ± 2.78 b	70.22 ± 7.48 b	75.26 ± 1.39 b	33.09 ± 2.19 c
	ProStab	9.67 ± 1.13	8.02 ± 1.04	9.64 ± 0.73	8.94 ± 0.77	8.19 ± 0.79
PR23	AFerm	133.92 ± 17.06 a	52.14 ± 1.53 b	37.56 ± 3.96 c	38.55 ± 1.41 c	15.12 ± 0.77 d
	ProStab	6.37 ± 0.82 a	4.86 ± 0.66 ab	4.67 ± 0.08 b	4.21 ± 0.41 b	2.93 ± 1.02 c
PR22	AFerm	63.77 ± 4.93 a	20.02 ± 1.10 b	10.86 ± 2.12 c	10.85 ± 0.89 c	3.98 ± 0.30 d
	ProStab	2.47 ± 0.56 a	2.17 ± 0.33 a	1.42 ± 0.16 b	1.14 ± 0.08 bc	0.68 ± 0.27 c
PR20	AFerm	76.03 ± 7.75 a	29.36 ± 1.86 b	21.70 ± 1.87 c	13.72 ± 0.11 d	4.89 ± 0.24 e
	ProStab	2.91 ± 0.11 a	2.59 ± 0.17 ab	2.69 ± 0.28 a	1.87 ± 0.28 c	1.99 ± 0.44 bc

TL—thaumatin-like proteins, CHI—chitinases, P93–PR20—proteins with numbers denominating the estimated molecular weight in kDa, CO—control wine without bentonite in fermentation, GSAB—initial dose (95 g/hL) of granular sodium-activated bentonite CX Special Grain added near the end of fermentation, PEN—initial dose (95 g/hL) of sodium-activated bentonite Pentagel added near the end of fermentation, MVN—initial dose (143 g/hL) of activated sodium bentonite with specifically adsorbed silica and activated silica Mastervin Compact added near the end of fermentation, PUR—initial dose (238 g/hL) of active sodium-calcium bentonite Siha Puranit bentonite added near the end of fermentation. AFerm—wines analyzed after fermentation, ProStab—wines analyzed after total protein stabilization by additional post-fermentation fining with granular sodium-activated bentonite CX Special Grain. ^1^ Concentrations are expressed as equivalents of thaumatin from *Thaumatococcus daniellii*. ^2^ Concentrations are expressed as equivalents of bovine serum albumin. Different lowercase letters in a row represent statistically significant differences among treatments, at *p* < 0.05 obtained by one-way ANOVA and LSD test.

## Supplementary Materials

The following are available online at https://www.mdpi.com/2304-8158/9/11/1534/s1, Table S1: Reduction of the concentrations of proteins in Malvazija istarska wines (mean ± standard deviation; *n* = 3; %) obtained after partial fining with bentonite at different points of fermentation and in final protein stable wines., Table S2: Reduction of the concentrations of proteins in Malvazija istarska wines (mean ± standard deviation; *n* = 3; %) obtained after partial fining with bentonite and/or the addition of commercial enological tannin preparation during fermentation, and in final protein stable wines, Table S3: Reduction of the concentrations of proteins in Malvazija istarska wines (mean ± standard deviation; *n* = 3; %) obtained after partial fining with different types of bentonite in fermentation, and in final protein stable wines, Figure S1: Total residual concentrations of pathogenesis-related (PR) proteins in protein stable Malvazija istarska wines (mean ± standard deviation; *n* = 3) obtained after partial fining with bentonite at different points of fermentation followed by additional fining by a required dose of granular sodium-activated bentonite after fermentation. CO—control wine without bentonite in fermentation, JU—initial granular sodium-activated bentonite dose added into clear juice, BE—initial granular sodium-activated bentonite dose added at the beginning of fermentation, MD—initial granular sodium-activated bentonite dose added in the middle of fermentation, EN—initial granular sodium-activated bentonite dose added near the end of fermentation. TL—thaumatin-like proteins, CHI—chitinases, RP-HPLC—reverse phase high-performance liquid chromatography, SE-HPLC—size exclusion high-performance liquid chromatography. Different lowercase letters above bars represent statistically significant differences among treatments with respect to total bentonite dose required, at *p* < 0.05 obtained by one-way ANOVA and LSD test, Figure S2: Total residual concentrations of pathogenesis-related (PR) proteins in protein stable Malvazija istarska wines (mean ± standard deviation; *n* = 3) obtained after partial fining with bentonite and/or the addition of commercial enological tannin preparation during fermentation followed by additional fining by a required dose of granular sodium-activated bentonite after fermentation. CO—control wine without bentonite or commercial enological tannin preparation added during fermentation, GSAB—initial dose of granular sodium-activated bentonite added near the end of fermentation, ET—commercial enological tannin preparation added during fermentation, GSAB + ET—initial dose of granular sodium-activated bentonite added near the end of fermentation and commercial enological tannin preparation added during fermentation. TL—thaumatin-like proteins, CHI—chitinases, RP-HPLC—reverse phase high-performance liquid chromatography, SE-HPLC—size exclusion high-performance liquid chromatography. Different lowercase letters above bars represent statistically significant differences among treatments with respect to total bentonite dose required, at *p* < 0.05 obtained by one-way ANOVA and LSD test, Figure S3: Percentage of bentonite sediment (%) after treatment of grape juice with a dose of 95 g/hL of granular sodium-activated bentonite and equivalent doses of other bentonites determined by a preliminary protein stability test. Abbreviations: GSAB—granular sodium-activated bentonite CX Special Grain, PEN—activated sodium bentonite Pentagel, MVN—activated sodium bentonite with specifically adsorbed silica and activated silica Mastervin Compact, PUR—active Na-Ca bentonite SIHA Puranit, Figure S4: Total residual concentrations of pathogenesis-related (PR) proteins in protein stable Malvazija istarska wines (mean ± standard deviation; *n* = 3) obtained after partial fining with different types of bentonite in fermentation followed by additional fining by a required dose of granular sodium-activated bentonite after fermentation. CO—control wine without bentonite in fermentation, GSAB—initial dose (95 g/hL) of granular sodium-activated bentonite CX Special Grain added near the end of fermentation, PEN—initial dose (95 g/hL) of sodium-activated bentonite Pentagel added near the end of fermentation, MVN—initial dose (143 g/hL) of activated sodium bentonite with specifically adsorbed silica and activated silica Mastervin Compact added near the end of fermentation, PUR—initial dose (238 g/hL) of active Na-Ca bentonite Siha Puranit bentonite added near the end of fermentation. TL—thaumatin-like proteins, CHI—chitinases, RP-HPLC—reverse phase high-performance liquid chromatography, SE-HPLC—size exclusion high-performance liquid chromatography. Different lowercase letters above bars represent statistically significant differences among treatments with respect to total bentonite dose required, at *p* < 0.05 obtained by one-way ANOVA and LSD test.
