# Supplementary material for: Moment of Bentonite Addition, Co-Addition of Tannins, and Bentonite Type Affect the Differential Affinity of Pathogenesis-Related Grape Proteins towards Bentonite during Fermentation
Source: Foods. 2020 Oct 25;9(11):1534. doi: 10.3390/foods9111534 (PMC7693156; doi:10.3390/foods9111534)
Supplement: Supplementary file 1 [file foods-09-01534-s001.pdf]

**Table S1.** Reduction of the concentrations of proteins in Malvazija istarska wines (mean ± standard deviation; n = 3; %) obtained after partial fining with bentonite at different points of fermentation and in final protein stable wines.

| Protein | Stage | Treatment | | | | |
|---|---|---|---|---|---|---|
| | | CO | JU | BE | MD | EN |
| *RP-HPLC* | | | | | | |
| TL1 | AFerm | | 52.17 ± 6.97 | 47.82 ± 20.75 | 56.08 ± 6.46 | 67.96 ± 8.96 |
| | ProStab | 97.48 ± 0.76 | 97.13 ± 0.53 | 97.41 ± 1.09 | 97.93 ± 1.08 | 95.80 ± 1.49 |
| TL2 | AFerm | | 32.27 ± 2.85 b | 33.56 ± 6.96 b | 35.23 ± 2.05 b | 52.52 ± 10.34 a |
| | ProStab | 85.85 ± 3.37 a | 89.53 ± 3.30 a | 89.95 ± 0.90 a | 89.50 ± 0.65 a | 76.69 ± 7.34 b |
| TL3 | AFerm | | 21.75 ± 4.45 | 18.90 ± 13.56 | 14.44 ± 5.01 | 32.41 ± 16.37 |
| | ProStab | 64.05 ± 8.04 b | 84.25 ± 5.57 a | 83.85 ± 0.36 a | 83.22 ± 2.69 a | 65.6 ± 11.46 b |
| TL4 | AFerm | | 58.87 ± 5.24 c | 60.91 ± 6.09 c | 87.69 ± 2.15 b | 96.61 ± 1.49 a |
| | ProStab | 98.57 ± 0.91 a | 97.41 ± 0.23 a | 97.80 ± 0.40 a | 89.89 ± 3.40 a | 59.86 ± 23.03 b |
| CHI1 | AFerm | | 48.27 ± 13.29 | 43.34 ± 16.77 | 44.55 ± 9.23 | 55.58 ± 8.27 |
| | ProStab | 98.7 ± 0.09 | 98.34 ± 0.31 | 97.78 ± 0.69 | 97.72 ± 0.36 | 97.38 ± 0.49 |
| CHI2 | AFerm | | 47.78 ± 11.36 | 47.03 ± 15.49 | 48.86 ± 9.20 | 61.87 ± 8.46 |
| | ProStab | 99.71 ± 0.04 | 99.38 ± 0.18 | 99.47 ± 0.19 | 99.41 ± 0.18 | 99.23 ± 0.26 |
| total TL | AFerm | | 48.61 ± 3.91 c | 47.86 ± 6.91 c | 61.36 ± 2.26 b | 73.48 ± 6.76 a |
| | ProStab | 92.39 ± 2.15 a | 93.47 ± 1.95 a | 93.71 ± 1.54 a | 91.13 ± 1.64 a | 80.29 ± 6.69 b |
| total CHI | AFerm | | 48.06 ± 12.45 | 44.95 ± 16.20 | 46.43 ± 9.20 | 58.33 ± 8.33 |
| | ProStab | 99.15 ± 0.03 | 98.79 ± 0.17 | 98.49 ± 0.49 | 98.43 ± 0.25 | 98.11 ± 0.42 |
| total | AFerm | | 48.37 ± 7.29 | 46.57 ± 10.85 | 54.74 ± 5.34 | 66.76 ± 7.27 |
| | ProStab | 95.37 ± 1.32 a | 95.77 ± 1.31 a | 95.83 ± 1.24 a | 94.92 ± 1.11 a | 90.34 ± 2.70 b |
| total TL / total CHI [1] | AFerm | | 1.05 ± 0.25 | 1.14 ± 0.32 | 1.35 ± 0.20 | 1.27 ± 0.10 |
| | ProStab | 0.93 ± 0.02 a | 0.95 ± 0.02 a | 0.95 ± 0.01 a | 0.93 ± 0.01 a | 0.82 ± 0.06 b |
| *SE-HPLC* | | | | | | |
| P93 | AFerm | | 26.63 ± 0.83 | 25.06 ± 7.74 | 18.62 ± 7.39 | 13.75 ± 4.78 |
| | ProStab | 20.68 ± 7.31 a | 6.06 ± 4.25 b | 4.59 ± 1.95 b | 12.96 ± 3.54 ab | 6.28 ± 6.24 b |
| P67 | AFerm | | 44.36 ± 6.95 | 54.98 ± 7.36 | 46.11 ± 2.92 | 53.85 ± 8.49 |
| | ProStab | 100.00 ± 0.00 | 100.00 ± 0.00 | 100.00 ± 0.00 | 100.00 ± 0.00 | 100.00 ± 0.00 |
| PR32 | AFerm | | 100.00 ± 0.00 | 100.00 ± 0.00 | 100.00 ± 0.00 | 100.00 ± 0.00 |
| | ProStab | 100.00 ± 0.00 | - | - | - | - |
| PR25 | AFerm | | 47.47 ± 4.63 | 54.48 ± 0.45 | 52.70 ± 1.44 | 55.46 ± 7.07 |
| | ProStab | 88.49 ± 3.72 c | 95.74 ± 1.87 a | 95.19 ± 0.33 ab | 95.83 ± 1.03 a | 91.32 ± 1.87 bc |
| PR23 | AFerm | | 31.21 ± 6.40 c | 46.19 ± 0.54 ab | 41.33 ± 0.70 bc | 51.89 ± 7.73 a |
| | ProStab | 89.65 ± 4.07 b | 96.61 ± 2.02 a | 96.75 ± 0.05 a | 96.72 ± 0.90 a | 90.50 ± 2.56 b |
| PR22 | AFerm | | 58.50 ± 5.08 c | 61.38 ± 4.78 c | 73.82 ± 2.04 b | 84.90 ± 2.83 a |
| | ProStab | 93.94 ± 2.24 a | 96.44 ± 1.75 a | 95.97 ± 0.43 a | 95.11 ± 1.58 a | 85.87 ± 1.91 b |
| PR20 | AFerm | | 53.55 ± 0.73 c | 58.37 ± 1.90 c | 67.79 ± 4.39 b | 74.34 ± 2.05 a |
| | ProStab | 84.80 ± 0.69 a | 79.38 ± 0.78 ab | 74.84 ± 3.44 bc | 74.74 ± 3.26 bc | 69.89 ± 4.44 c |
| total PR | AFerm | | 48.99 ± 3.62 c | 55.85 ± 1.40 bc | 59.01 ± 0.82 b | 65.71 ± 5.08 a |
| | ProStab | 89.47 ± 2.61 ab | 93.46 ± 1.81 a | 92.30 ± 0.47 a | 93.04 ± 1.29 a | 87.71 ± 2.48 b |

CO – control wine without bentonite in fermentation, JU – initial granular sodium-activated bentonite dose (100 g/hL) added into clear juice, BE – initial granular sodium-activated bentonite dose (100 g/hL) added at the beginning of fermentation, MD – initial granular sodium-activated bentonite dose (100 g/hL) added in the middle of fermentation, EN – initial granular sodium-activated bentonite dose (100 g/hL) added near the end of fermentation. AFerm – wines analyzed after fermentation, ProStab – wines analyzed after total protein stabilization by additional post-fermentation fining with granular sodium-activated bentonite. AFerm reductions were calculated in relation to CO wine and ProStab reductions in relation to corresponding AFerm wines. Different lowercase letters in a row represent statistically significant differences among treatments, at $p < 0.05$ obtained by one-way ANOVA and LSD test. [1] ratio of % values.

**Table S2.** Reduction of the concentrations of proteins in Malvazija istarska wines (mean ± standard deviation; n = 3; %) obtained after partial fining with bentonite and/or the addition of commercial enological tannin preparation during fermentation, and in final protein stable wines.

| Protein | Stage | Treatment | | | |
|---|---|---|---|---|---|
| | | CO | GSAB | ET | GSAB + ET |
| *RP-HPLC* | | | | | |
| TL1 | AFerm | | 78.20 ± 4.49 a | 23.82 ± 9.19 b | 89.51 ± 3.51 a |
| | ProStab | 96.04 ± 4.98 | 88.48 ± 8.88 | 95.21 ± 3.20 | 92.43 ± 2.89 |
| TL2 | AFerm | | 60.50 ± 2.95 a | 18.26 ± 8.16 b | 71.36 ± 6.65 a |
| | ProStab | 86.09 ± 5.95 a | 65.81 ± 6.45 b | 85.25 ± 1.31 a | 56.34 ± 10.88 b |
| TL3 | AFerm | | 31.10 ± 1.83 | 11.27 ± 14.76 | 30.02 ± 9.68 |
| | ProStab | 72.18 ± 8.23 a | 49.08 ± 7.62 b | 71.23 ± 7.18 a | 56.14 ± 7.82 b |
| TL4 | AFerm | | 97.59 ± 1.77 a | 19.90 ± 5.59 b | 99.04 ± 0.62 a |
| | ProStab | 97.53 ± 2.93 | 100.00 ± 0.00 | 95.06 ± 3.53 | 89.07 ± 18.93 |
| CHI1 | AFerm | | 52.45 ± 5.93 a | 5.40 ± 15.22 b | 71.37 ± 3.46 a |
| | ProStab | 90.13 ± 7.33 | 73.45 ± 9.64 | 88.65 ± 5.73 | 80.05 ± 7.93 |
| CHI2 | AFerm | | 54.08 ± 7.16 a | -0.56 ± 16.86 b | 72.82 ± 2.98 a |
| | ProStab | 90.80 ± 5.86 a | 70.99 ± 7.17 b | 88.34 ± 4.86 a | 79.11 ± 7.57 ab |
| total TL | AFerm | | 74.90 ± 2.83 b | 19.97 ± 3.26 c | 81.25 ± 3.37 a |
| | ProStab | 91.58 ± 5.06 ab | 67.86 ± 5.65 | 89.97 ± 2.18 ab | 64.82 ± 5.69 b |
| total CHI | AFerm | | 53.20 ± 6.48 a | 2.65 ± 15.97 b | 72.04 ± 3.12 a |
| | ProStab | 90.44 ± 6.65 a | 72.35 ± 8.55 b | 88.50 ± 5.26 a | 79.60 ± 7.61 ab |
| total | AFerm | | 65.90 ± 4.29 b | 12.78 ± 6.94 c | 77.43 ± 3.19 a |
| | ProStab | 91.10 ± 5.72 a | 70.40 ± 7.21 b | 89.33 ± 3.44 a | 72.48 ± 5.48 b |
| total TL / total CHI [1] | AFerm | | 1.42 ± 0.12 | 0.90 ± 2.03 | 1.13 ± 0.02 |
| | ProStab | 1.01 ± 0.02 a | 0.94 ± 0.04 a | 1.02 ± 0.04 a | 0.82 ± 0.08 b |
| *SE-HPLC* | | | | | |
| P93 | AFerm | | 3.94 ± 0.34 | 15.05 ± 13.26 | 13.38 ± 15.78 |
| | ProStab | -6.41 ± 10.96 | 2.41 ± 2.81 | -29.95 ± 29.80 | -25.67 ± 24.76 |
| P67 | AFerm | | 38.57 ± 1.52 | 35.69 ± 6.04 | 60.24 ± 21.02 |
| | ProStab | 88.57 ± 9.82 | 80.79 ± 7.15 | 93.74 ± 10.84 | 100.00 ± 0.00 |
| PR32 | AFerm | | 100.00 ± 0.00 a | 29.29 ± 8.81 b | 100.00 ± 0.00 a |
| | ProStab | 100.00 ± 0.00 | - | 100.00 ± 0.00 | - |
| PR25 | AFerm | | 52.52 ± 5.80 b | 24.43 ± 1.32 c | 73.59 ± 2.73 a |
| | ProStab | 88.55 ± 7.08 a | 71.95 ± 4.63 b | 89.94 ± 2.80 a | 70.75 ± 7.46 b |
| PR23 | AFerm | | 61.31 ± 5.36 b | 29.87 ± 3.20 c | 81.36 ± 4.73 a |
| | ProStab | 91.16 ± 5.54 a | 79.96 ± 4.06 b | 92.81 ± 3.17 a | 75.32 ± 8.73 b |
| PR22 | AFerm | | 82.18 ± 2.71 b | 16.96 ± 5.58 c | 92.76 ± 2.69 a |
| | ProStab | 92.05 ± 4.61 ab | 84.34 ± 4.71 b | 98.94 ± 1.30 a | 93.80 ± 6.18 a |
| PR20 | AFerm | | 75.72 ± 1.19 a | 22.06 ± 4.00 b | 79.89 ± 4.07 a |
| | ProStab | 81.62 ± 1.43 a | 51.50 ± 0.74 b | 83.67 ± 1.28 a | 19.65 ± 13.65 c |
| total PR | AFerm | | 65.50 ± 3.99 b | 23.96 ± 2.09 c | 80.37 ± 3.34 a |
| | ProStab | 88.33 ± 4.90 a | 71.61 ± 3.42 b | 90.84 ± 1.59 a | 61.06 ± 6.65 c |

CO – control wine without bentonite or commercial enological tannin preparation added during fermentation, GSAB – initial dose (95 g/hL) of granular sodium-activated bentonite added near the end of fermentation, ET – commercial enological tannin preparation (25 g/hL divided in three portions) added during fermentation, GSAB + ET – initial dose (95 g/hL) of granular sodium-activated bentonite added near the end of fermentation and commercial enological tannin preparation (25 g/hL divided in three portions) added during fermentation. AFerm – wines analyzed after fermentation, ProStab – wines analyzed after total protein stabilization by additional post-fermentation fining with bentonite. AFerm reductions were calculated in relation to CO wine and ProStab reductions in relation to corresponding AFerm wines. Different lowercase letters in a row represent statistically significant differences among treatments, at $p < 0.05$ obtained by one-way ANOVA and LSD test. [1] ratio of % values.

**Table S3.** Reduction of the concentrations of proteins in Malvazija istarska wines (mean ± standard deviation; n = 3; %) obtained after partial fining with different types of bentonite in fermentation, and in final protein stable wines.

| Protein | Stage | Treatment | | | | |
|---|---|---|---|---|---|---|
| | | CO | GSAB | PEN | MVN | PUR |
| *RP-HPLC* | | | | | | |
| TL1 | AFerm | | 66.94 ± 2.24 c | 74.72 ± 3.30 b | 73.25 ± 1.86 b | 94.16 ± 1.10 a |
| | ProStab | 95.76 ± 0.92 a | 88.65 ± 0.41 bc | 86.09 ± 1.44 bc | 89.47 ± 0.93 b | 84.58 ± 3.56 c |
| TL2 | AFerm | | 49.70 ± 3.21 b | 48.83 ± 2.26 b | 46.55 ± 2.87 b | 73.95 ± 2.08 a |
| | ProStab | 93.18 ± 0.39 a | 87.07 ± 1.03 b | 80.94 ± 3.79 c | 84.61 ± 2.03 bc | 74.11 ± 2.7 d |
| TL3 | AFerm | | 28.64 ± 5.21 b | 13.35 ± 5.27 c | 10.70 ± 4.31 c | 49.28 ± 2.45 a |
| | ProStab | 88.43 ± 0.99 a | 84.23 ± 1.47 ab | 77.30 ± 5.15 b | 80.74 ± 4.27 b | 64.66 ± 2.45 c |
| TL4 | AFerm | | 77.38 ± 1.20 b | 96.83 ± 1.94 a | 97.76 ± 0.34 a | 98.49 ± 0.17 a |
| | ProStab | 97.27 ± 1.16 | 91.46 ± 0.57 | 91.49 ± 1.49 | 93.44 ± 1.59 | 95.54 ± 5.30 |
| CHI1 | AFerm | | 62.84 ± 3.22 c | 70.30 ± 3.11 b | 61.80 ± 3.30 c | 83.41 ± 1.77 a |
| | ProStab | 94.97 ± 0.21 a | 88.70 ± 0.62 b | 85.54 ± 1.85 b | 88.52 ± 0.47 b | 76.74 ± 3.62 c |
| CHI2 | AFerm | | 63.13 ± 3.37 c | 74.78 ± 3.09 b | 66.61 ± 2.78 c | 85.78 ± 1.44 a |
| | ProStab | 95.34 ± 0.30 a | 88.91 ± 0.74 b | 85.18 ± 2.04 b | 87.87 ± 0.47 b | 77.69 ± 4.21 c |
| total TL | AFerm | | 64.87 ± 1.48 c | 73.39 ± 2.32 b | 72.52 ± 1.55 b | 89.09 ± 1.00 a |
| | ProStab | 95.29 ± 0.77 a | 88.16 ± 0.66 b | 82.45 ± 3.10 c | 85.85 ± 1.80 bc | 73.56 ± 2.9 d |
| total CHI | AFerm | | 62.97 ± 3.29 c | 72.30 ± 3.09 b | 63.96 ± 3.07 c | 84.47 ± 1.62 a |
| | ProStab | 95.14 ± 0.24 a | 88.79 ± 0.67 b | 85.39 ± 1.90 b | 88.25 ± 0.39 b | 77.13 ± 3.86 c |
| total | AFerm | | 63.94 ± 2.36 c | 72.86 ± 2.70 b | 68.34 ± 2.14 c | 86.84 ± 1.30 a |
| | ProStab | 95.22 ± 0.52 a | 88.48 ± 0.67 b | 83.91 ± 2.47 c | 87.18 ± 0.91 bc | 75.61 ± 3.39 d |
| total TL / total CHI [1] | AFerm | | 1.03 ± 0.03 b | 1.02 ± 0.01 b | 1.14 ± 0.04 a | 1.05 ± 0.01 b |
| | ProStab | 1.00 ± 0.01 | 0.99 ± 0.00 | 0.97 ± 0.02 | 0.97 ± 0.02 | 0.95 ± 0.02 |
| *SE-HPLC* | | | | | | |
| P93 | AFerm | | 20.00 ± 4.92 | 21.53 ± 11.26 | 24.96 ± 1.36 | 30.11 ± 5.22 |
| | ProStab | 11.45 ± 17.63 | 4.28 ± 14.61 | 6.07 ± 10.06 | 5.47 ± 6.51 | 3.35 ± 9.35 |
| P67 | AFerm | | 10.23 ± 6.27 b | 2.14 ± 6.70 b | -2.16 ± 8.44 b | 41.29 ± 3.74 a |
| | ProStab | 89.02 ± 0.36 a | 86.31 ± 0.05 a | 81.33 ± 1.87 a | 83.27 ± 3.82 a | 68.53 ± 11.63 b |
| PR32 | AFerm | | 100.00 ± 0.00 | 100.00 ± 0.00 | 100.00 ± 0.00 | 100.00 ± 0.00 |
| | ProStab | 100.00 ± 0.00 | - | - | - | - |
| PR25 | AFerm | | 52.71 ± 1.53 c | 61.30 ± 4.12 b | 58.52 ± 0.77 b | 81.76 ± 1.21 a |
| | ProStab | 94.59 ± 0.66 a | 90.64 ± 1.52 ab | 86.10 ± 2.52 b | 88.11 ± 1.23 b | 75.06 ± 4.12 c |
| PR23 | AFerm | | 61.07 ± 1.14 c | 71.96 ± 2.96 b | 71.21 ± 1.05 b | 88.71 ± 0.57 a |
| | ProStab | 95.24 ± 0.01 a | 90.66 ± 1.54 a | 87.46 ± 1.33 ab | 89.06 ± 1.33 a | 80.45 ± 7.59 b |
| PR22 | AFerm | | 68.60 ± 1.72 c | 82.96 ± 3.33 b | 82.99 ± 1.39 b | 93.76 ± 0.47 a |
| | ProStab | 96.15 ± 0.58 | 89.18 ± 1.06 | 86.75 ± 1.82 | 89.45 ± 1.52 | 82.43 ± 8.46 |
| PR20 | AFerm | | 61.38 ± 2.45 d | 71.46 ± 2.46 c | 81.96 ± 0.15 b | 93.57 ± 0.31 a |
| | ProStab | 96.15 ± 0.25 a | 91.18 ± 0.03 a | 87.54 ± 1.85 a | 86.37 ± 2.09 a | 59.01 ± 10.96 b |
| total PR | AFerm | | 59.20 ± 0.29 c | 69.44 ± 3.23 b | 69.87 ± 0.70 b | 87.57 ± 0.72 a |
| | ProStab | 95.32 ± 0.18 a | 90.58 ± 1.24 ab | 86.73 ± 2.02 b | 88.31 ± 1.31 b | 75.65 ± 4.89 c |

CO – control wine without bentonite in fermentation, GSAB – initial dose (95 g/hL) of granular sodium-activated CX Special Grain bentonite added near the end of fermentation, PEN – initial dose (95 g/hL) of Pentagel bentonite added near the end of fermentation, MVN – initial dose (143 g/hL) of Mastervin Compact bentonite added near the end of fermentation, PUR – initial dose (238 g/hL) of Siha Puranit bentonite added near the end of fermentation. AFerm – wines analyzed after fermentation, ProStab – wines analyzed after total protein stabilization by additional post-fermentation fining with granular sodium-activated CX Special Grain bentonite. AFerm reductions were calculated in relation to CO wine and ProStab reductions in relation to corresponding AFerm wines. Different lowercase letters in a row represent statistically significant differences among treatments, at $p < 0.05$ obtained by one-way ANOVA and LSD test. [1] ratio of % values.

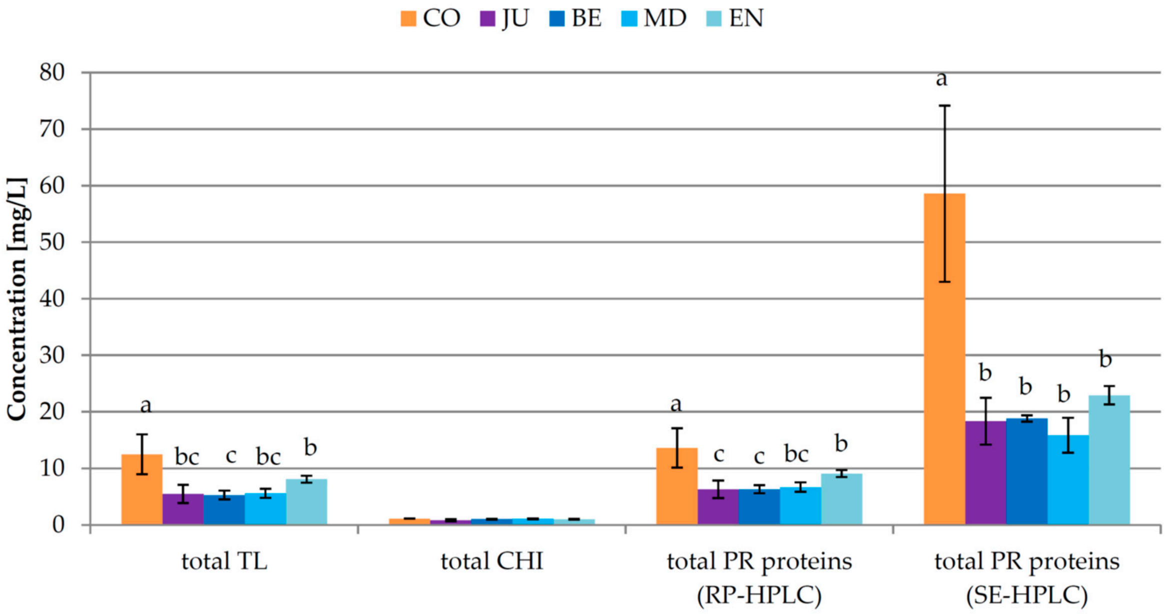


**Figure S1.** Total residual concentrations of pathogenesis-related (PR) proteins in protein stable Malvazija istarska wines (mean ± standard deviation; n = 3) obtained after partial fining with bentonite at different points of fermentation followed by additional fining by a required dose of granular sodium-activated bentonite after fermentation. CO – control wine without bentonite in fermentation, JU – initial granular sodium-activated bentonite dose added into clear juice, BE – initial granular sodium-activated bentonite dose added at the beginning of fermentation, MD – initial granular sodium-activated bentonite dose added in the middle of fermentation, EN – initial granular sodium-activated bentonite dose added near the end of fermentation. TL – thaumatin-like proteins, CHI – chitinases, RP-HPLC – reverse phase high-performance liquid chromatography, SE-HPLC – size exclusion high-performance liquid chromatography. Different lowercase letters above bars represent statistically significant differences among treatments with respect to total bentonite dose required, at $p < 0.05$ obtained by one-way ANOVA and LSD test.

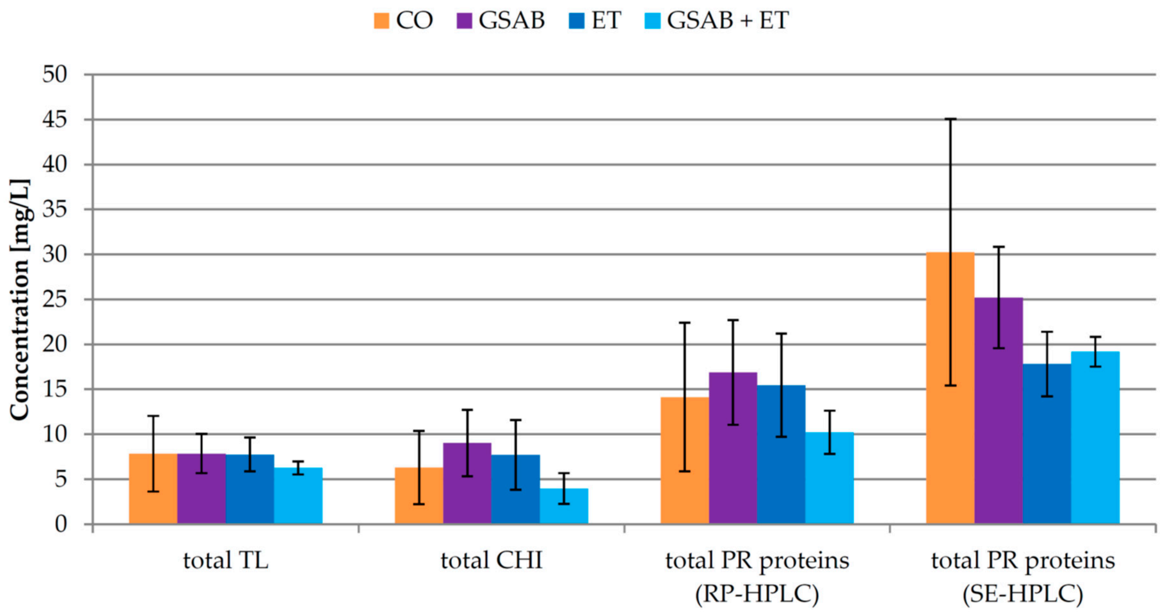


**Figure S2.** Total residual concentrations of pathogenesis-related (PR) proteins in protein stable Malvazija istarska wines (mean ± standard deviation; n = 3) obtained after partial fining with bentonite and/or the addition of commercial enological tannin preparation during fermentation followed by additional fining by a required dose of granular sodium activated bentonite after fermentation. CO – control wine without bentonite or commercial enological tannin preparation added during fermentation, GSAB – initial dose of granular sodium-activated bentonite added near the end of fermentation, ET – commercial enological tannin preparation added during fermentation, GSAB + ET – initial dose of granular sodium-activated bentonite added near the end of fermentation and commercial enological tannin preparation added during fermentation. TL – thaumatin-like proteins, CHI – chitinases, RP-HPLC – reverse phase high-performance liquid chromatography, SE-HPLC – size exclusion high-performance liquid chromatography. Different lowercase letters above bars represent statistically significant differences among treatments with respect to total bentonite dose required, at $p < 0.05$ obtained by one-way ANOVA and LSD test,

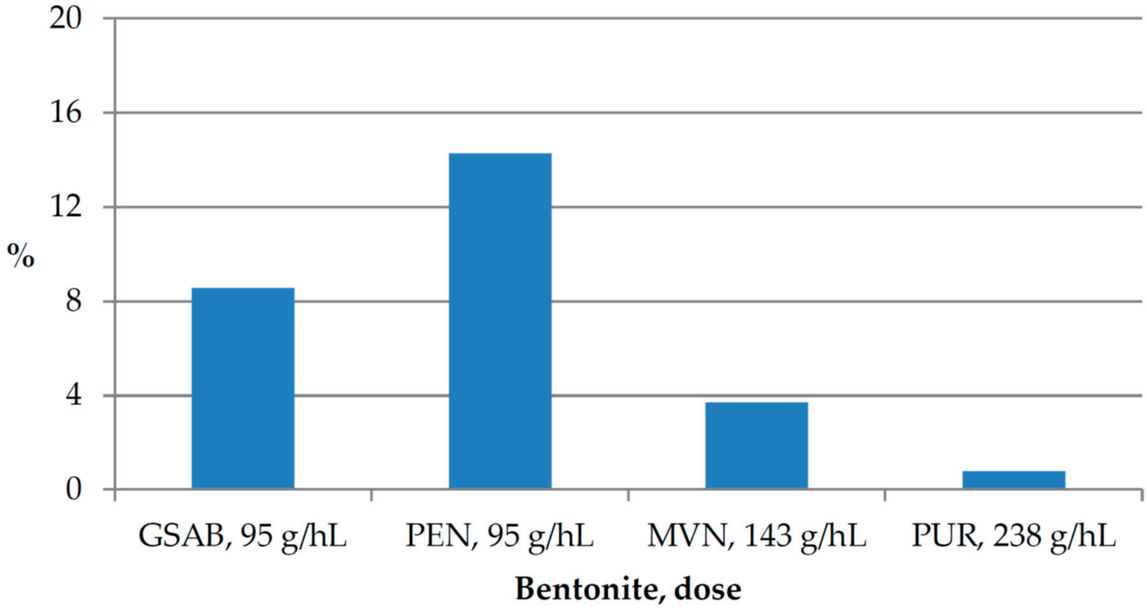


**Figure S3.** Percentage of bentonite sediment (%) after treatment of grape juice with a dose of 95 g/hL of granular sodium-activated bentonite and equivalent doses of other bentonites determined by a preliminary protein stability test. Abbreviations: GSAB - granular sodium-activated bentonite CX Special Grain, PEN - activated sodium bentonite Pentagel, MVN - activated sodium bentonite with specifically adsorbed silica and activated silica Mastervin Compact, PUR - active Na-Ca bentonite SIHA Puranit

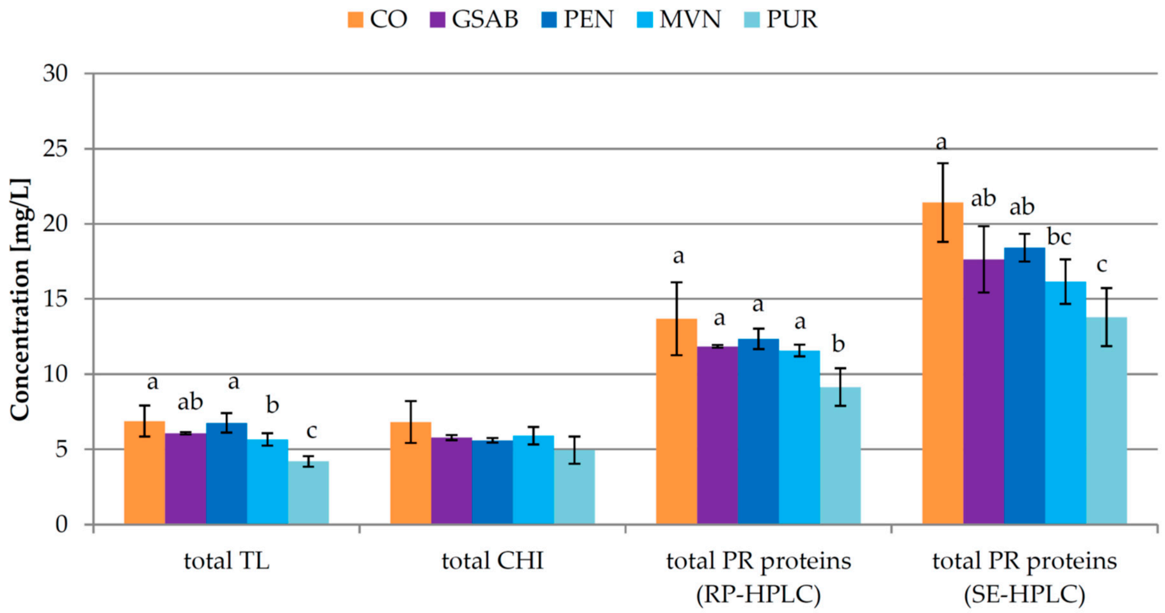


**Figure S4.** Total residual concentrations of pathogenesis-related (PR) proteins in protein stable Malvazija istarska wines (mean ± standard deviation; n = 3) obtained after partial fining with different types of bentonite in fermentation followed by additional fining by a required dose of granular sodium-activated bentonite after fermentation. CO – control wine without bentonite in fermentation, GSAB – initial dose (95 g/hL) of granular sodium-activated bentonite CX Special Grain added near the end of fermentation, PEN – initial dose (95 g/hL) of sodium-activated bentonite Pentagel added near the end of fermentation, MVN – initial dose (143 g/hL) of activated sodium bentonite with specifically adsorbed silica and activated silica Mastervin Compact added near the end of fermentation, PUR – initial dose (238 g/hL) of active Na-Ca bentonite Siha Puranit bentonite added near the end of fermentation. TL – thaumatin-like proteins, CHI – chitinases, RP-HPLC – reverse phase high-performance liquid chromatography, SE-HPLC – size exclusion high-performance liquid chromatography. Different lowercase letters above bars represent statistically significant differences among treatments with respect to total bentonite dose required, at $p < 0.05$ obtained by one-way ANOVA and LSD test.
